# A pre-post quasi-experimental study of the impact of TDM-guided aggressive pharmacokinetic/pharmacodynamic target attainment of continuous infusion ceftolozane/tazobactam monotherapy in treating severe *Pseudomonas aeruginosa* infections: a strategy useful for raising the bar?

**DOI:** 10.1093/jac/dkaf098

**Published:** 2025-03-24

**Authors:** Milo Gatti, Matteo Rinaldi, Cecilia Bonazzetti, Antonio Siniscalchi, Tommaso Tonetti, Simone Ambretti, Maddalena Giannella, Pierluigi Viale, Federico Pea

**Affiliations:** Department of Medical and Surgical Sciences, Alma Mater Studiorum University of Bologna, Bologna, Italy; Clinical Pharmacology Unit, Department for Integrated Infectious Risk Management, IRCCS Azienda Ospedaliero-Universitaria di Bologna, Via Massarenti 9, Bologna 40138, Italy; Department of Medical and Surgical Sciences, Alma Mater Studiorum University of Bologna, Bologna, Italy; Infectious Diseases Unit, Department for Integrated Infectious Risk Management, IRCCS Azienda Ospedaliero-Universitaria di Bologna, Bologna, Italy; Department of Medical and Surgical Sciences, Alma Mater Studiorum University of Bologna, Bologna, Italy; Infectious Diseases Unit, Department for Integrated Infectious Risk Management, IRCCS Azienda Ospedaliero-Universitaria di Bologna, Bologna, Italy; Anesthesia and Intensive Care Medicine, IRCCS Azienda Ospedaliero-Universitaria di Bologna, Bologna, Italy; Department of Medical and Surgical Sciences, Alma Mater Studiorum University of Bologna, Bologna, Italy; Division of Anesthesiology, Department of Anesthesia and Intensive Care, IRCCS Azienda Ospedaliero-Universitaria di Bologna, Bologna, Italy; Department of Medical and Surgical Sciences, Alma Mater Studiorum University of Bologna, Bologna, Italy; Operative Unit of Microbiology, Department for Integrated Infectious Risk Management, IRCCS Azienda Ospedaliero-Universitaria di Bologna, Bologna, Italy; Department of Medical and Surgical Sciences, Alma Mater Studiorum University of Bologna, Bologna, Italy; Infectious Diseases Unit, Department for Integrated Infectious Risk Management, IRCCS Azienda Ospedaliero-Universitaria di Bologna, Bologna, Italy; Department of Medical and Surgical Sciences, Alma Mater Studiorum University of Bologna, Bologna, Italy; Infectious Diseases Unit, Department for Integrated Infectious Risk Management, IRCCS Azienda Ospedaliero-Universitaria di Bologna, Bologna, Italy; Department of Medical and Surgical Sciences, Alma Mater Studiorum University of Bologna, Bologna, Italy; Clinical Pharmacology Unit, Department for Integrated Infectious Risk Management, IRCCS Azienda Ospedaliero-Universitaria di Bologna, Via Massarenti 9, Bologna 40138, Italy

## Abstract

**Objectives:**

To assess the clinical usefulness of a therapeutic drug monitoring (TDM)-guided strategy for attaining an aggressive pharmacokinetic/pharmacodynamic (PK/PD) target of continuous infusion (CI) ceftolozane/tazobactam monotherapy in patients having *Pseudomonas aeruginosa* infections.

**Methods:**

We performed a pre-post quasi-experimental study including adult patients with documented *P. aeruginosa* bacteraemia and/or pneumonia who were treated with CI ceftolozane/tazobactam monotherapy tailored by means of a TDM-guided strategy in the period 1 November 2023 to 31 July 2024 (post-intervention phase) compared with patients receiving standard management with CI ceftolozane/tazobactam monotherapy in the period 1 April 2022 to 31 October 2023 (pre-intervention phase). Clinical outcomes were compared between pre- and post-intervention phase. Univariate and multivariate analyses were performed for identifying variables associated with microbiological failure.

**Results:**

Eighty-five patients (48 in pre- and 37 in post-intervention phase) were included. Demographics and clinical features were similar in both groups. No significant difference emerged between groups in terms of microbiological eradication (*P* = 0.10), 30 day resistance to ceftolozane/tazobactam (*P* = 0.37), clinical cure (*P* = 0.26) and 30 day mortality rate (*P* = 0.79). All patients in the post-intervention phase attained an optimal PK/PD target, allowing the use of a lower ceftolozane/tazobactam CI daily dosing regimen compared with the pre-intervention phase (3.0 g/1.5 g versus 6.0 g/3.0 g; *P* = 0.06). The only independent predictors of microbiological failure were difficult-to-treat resistant *P. aeruginosa* isolates in the pre-intervention group (OR 6.99; 95% CI 1.34–36.55), and a ratio of partial pressure of arterial oxygen to fraction of oxygen in the inhaled air (Pao_2_/Fio_2_ ratio) <200 in the post-intervention group (OR 18.00; 95% CI 1.86–174.22).

**Conclusions:**

Our TDM-guided strategy of CI ceftolozane/tazobactam was cost-effective in attaining an aggressive PK/PD target of ceftolozane against susceptible *P. aeruginosa* strains with lower than standard daily doses without compromising efficacy.

## Introduction

*Pseudomonas aeruginosa* is one of the major pathogens causing healthcare-associated infections in critically ill and/or immunocompromised patients.^1,2^ More than 25% of ventilator-associated pneumonia (VAP) and approximately 15% of bloodstream infections (BSIs) occurring in the ICU setting are caused by *P. aeruginosa*.^1,2^ Unfortunately, a number of reasons make choosing the appropriate treatment for *P. aeruginosa*–related infections challenging, namely their opportunistic nature, the presence of multiple virulence mechanisms, and the capability to produce resistance against most antibiotics, as in the case of the so-called difficult-to-treat resistant (DTR) *P. aeruginosa*.^2^

Ceftolozane/tazobactam is a novel β-lactam/β-lactamase inhibitor combination currently considered by several international guidelines and/or guidance as first-line treatment of DTR *P. aeruginosa* infections.^3–5^ Indeed, ceftolozane *per se* has excellent *in vitro* activity against DTR *P. aeruginosa*,^2,6^ and does not require tazobactam to restore activity, irrespective of several resistance mechanisms.^7^ An early *in vitro* study testing ceftolozane activity both alone and in combination with tazobactam against 449 strains of *P. aeruginosa* with different resistance phenotypes showed that tazobactam addition did not lead to any significant enhancement of ceftolozane activity.^7^ Consequently, when assessing the efficacy of ceftolozane/tazobactam in the treatment of documented *P. aeruginosa* infections it makes sense to take into account only the pharmacokinetic/pharmacodynamic (PK/PD) determinant of ceftolozane activity, namely the time for which ceftolozane free concentrations persist above the MIC of *P. aeruginosa* (*f*T_>MIC_).^8^

A multidisciplinary international position paper stated recently that therapeutic drug monitoring (TDM) should be considered the only effective way for using antibiotics in critically ill patients.^9^ In this regard, a recent meta-analysis showed that the TDM-guided approach to traditional β-lactams in critically ill patients leads either to higher likelihood of optimal PK/PD target attainment or to better clinical cure and microbiological eradication rates compared with the standard approach.^10^ Indeed, the definition of optimal PK/PD target attainment for β-lactams is currently undergoing a paradigm shift in critically ill patients, with debate about whether targeting higher PK/PD thresholds could be of further benefit.^11^ In this scenario, a recent meta-analysis showed that attaining an aggressive PK/PD target of at least 100%*f*T_ > 4×MIC_ with β-lactams in critically ill patients may grant both better clinical efficacy and higher microbiological eradication rate compared with a conservative PK/PD target of 100%*f*T_>MIC_ in the treatment of Gram-negative documented infections, and may also minimize resistance occurrence.^12^ Alternative dosing regimens based on a loading dose followed by a CI maintenance dose (MD) may represent the most effective strategy for maximizing the likelihood of attaining aggressive PK/PD targets (namely free steady-state concentration [*fC*_ss_]/MIC ratio  >4, equivalent to 100%*f*T_>4×MIC_) under the same daily dose,^13^ and this strategy was recently reported also for ceftolozane/tazobactam.^14–16^

Based on these assumptions, it could be hypothesized that attaining an aggressive PK/PD target with TDM-guided CI ceftolozane/tazobactam could be helpful in maximizing clinical outcomes of severe *P. aeruginosa* infections in critically ill patients treated with ceftolozane/tazobactam monotherapy.

The aim of this study was to assess, by means of a pre-post quasi-experimental design, the impact that a TDM-guided approach focused on aggressive PK/PD target attainment with ceftolozane/tazobactam monotherapy could have on the clinical outcome of severe *P. aeruginosa* bloodstream infections (BSIs) and/or pneumonia.

## Methods

This retrospective pre-post quasi-experimental study included adult patients with documented *P. aeruginosa* BSIs and/or pneumonia who in the period 1 April 2022 to 31 July 2024 were treated with CI ceftolozane/tazobactam monotherapy at the IRCCS Azienda Ospedaliero-Universitaria of Bologna, Italy. Patients included in the pre-intervention phase (1 April 2022 to 31 October 2023) received treatment of documented *P. aeruginosa* BSIs and/or pneumonia with standard CI ceftolozane/tazobactam monotherapy. Patients included in the post-intervention phase (1 November 2023 to 31 July 2024) received treatment of the same type of infections with CI ceftolozane/tazobactam monotherapy tailored by means of a TDM-guided expert clinical pharmacological advice (ECPA) programme. Specifically, the ECPA was structured as an expert interpretation of each TDM result provided by the Doctor of Medicine (MD) clinical pharmacologist taking care of the site of infection, the *in vitro* susceptibility of the clinical isolate, the patient’s underlying conditions and/or the eventual need for renal support [i.e. continuous renal replacement therapy (CRRT) or intermittent haemodialysis]. Any type of recommendation (namely, dosage confirmed, decreased or increased) was usually based on expert opinion.^17^ This latter service became available for ceftolozane/tazobactam in November 2023 as an integral part of the programme originally started at our tertiary university hospital in 2021 and already covering several other antimicrobials.^17^ The presence of an infection control programme and an on-demand infectious diseases consultant service led by the same specialists in both phases granted consistent clinical expertise in the management of severe *P. aeruginosa* infections. The study was approved by the Ethics Committee of IRCCS Azienda Ospedaliero-Universitaria of Bologna (no. EM 232–2022_308/2021/Oss/AOUBo on 16 March 2022).

For cases in both pre- and post-intervention phases we collected: (i) demographic data—age, sex, BMI; (ii) clinical/laboratory data—underlying diseases, admission ward, SOFA score at the start of ceftolozane/tazobactam therapy, requirement for vasopressor support, need for mechanical ventilation, baseline partial pressure of oxygen to fraction of oxygen in the inhaled air ratio (Pao_2_/Fio_2_), baseline CL_CR_ estimated by means of the CKD-EPI formula,^18^ requirement for CRRT, occurrence of augmented renal clearance (ARC, defined as a normal serum creatinine level coupled with an estimated CL_CR_  >130 mL/min/1.73 m^2^ in males and >120 mL/min/1.73 m^2^ in females);^19^ (iii) microbiological data—type of infection, *P. aeruginosa* susceptibility and MIC value of ceftolozane/tazobactam; (iv) treatment data—ceftolozane/tazobactam dosing regimen, implementation of a TDM-guided ECPA approach, plasma concentrations, treatment duration; and (v) outcome data—microbiological eradication, 30 day resistance occurrence, clinical cure and 30 day mortality.

CDC criteria were adopted for defining the different types of infection.^20^ Documented BSI was defined based on the isolation of *P. aeruginosa* from at least one of two blood cultures carried out from different sites.^20^ Documented hospital-acquired pneumonia (HAP) and ventilator-associated pneumonia (VAP) were defined based on the isolation of *P. aeruginosa* with a bacterial load ≥10^4^ cfu/mL from the bronchoalveolar lavage fluid culture or with a bacterial load ≥10^6^ cfu/mL from the endotracheal aspirate after at least 48 h from hospital admission or from endotracheal intubation, respectively.^21^ Late-onset VAP was defined as the development of VAP after 4 days of intubation and mechanical ventilation.^22^

*P. aeruginosa* clinical isolates were identified by means of MALDI-TOF MS using the Maldi Biotyper Sirius system (Bruker Daltonics, Germany). Isolates were defined as being multi-susceptible when having a WT profile (namely, susceptible to all the first-line anti-pseudomonal agents, i.e. ceftazidime, cefepime, fluoroquinolones, aminoglycosides, carbapenems and piperacillin/tazobactam), or DTR when having an intermediate or resistant profile to all the first-line anti-pseudomonal agents.^23,24^

The broth microdilution method (panel provided by ITGN Merlin Diagnostika GMBH, Bornheim-Hersel, Germany) was used for testing ceftolozane/tazobactam susceptibility. MIC values of ceftolozane/tazobactam ranging from 0.5 to 64 mg/L were tested in the presence of a fixed target tazobactam concentration of 4 mg/L, and interpreted according to the EUCAST guidelines.^25^  *P. aeruginosa* strains having an MIC value  >4 mg/L were defined as resistant to ceftolozane/tazobactam.

In both phases, ceftolozane/tazobactam was used as first-line therapy for treating documented DTR *P. aeruginosa* infections or as rescue therapy for treating documented multi-susceptible *P. aeruginosa* infections failing first-line therapy with piperacillin/tazobactam, ceftazidime, cefepime or carbapenems. Specifically, failure of first-line anti-pseudomonal therapy was defined as the lack of improvement/resolution of signs and symptoms of the infection after at least 7 days of active therapy or the occurrence of breakthrough BSI and/or HAP/VAP. Therapy was always started with a loading dose of 2 g/1 g over 2 h, immediately followed by a CI MD selected on the basis of the patient’s estimated CL_CR_ (6 g/3 g if CL_CR_ >50 mL/min/1.73 m^2^; 3 g/1.5 g if CL_CR_ 30–50 mL/min/1.73 m^2^; or in case of CRRT, 1.5 g/0.75 g if CL_CR_ 15–29 mL/min/1.73 m^2^, and 1 g/0.5 g if CL_CR_ <15 mL/min/1.73 m^2^). Aqueous solutions were reconstituted every 24 h and infused over 24 h thanks to the well-established long-term stability at room temperature.^26^

In the pre-intervention phase, dosing adjustments during treatment were provided only if patients had fluctuations in renal function. In the post-intervention phase, ceftolozane/tazobactam therapy was tailored by means of a real-time TDM-guided ECPA programme.^27^ The first TDM-guided ECPA was performed after at least 24 h from starting therapy, and reassessed every 48–72 h whenever feasible. TDM sessions were carried out twice or thrice weekly with a turnaround time of 24–72 h depending on timing of sample delivery in the laboratory. Total ceftolozane steady-state plasma concentrations (*C*_ss_) were determined by means of a validated LC tandem MS method (lower and upper limits of quantification for ceftolozane were 0.2 mg/L and 200 mg/L, respectively).^28^ The free fraction (*f*) of ceftolozane was calculated by multiplying the total *C*_ss_ by 0.80, based on an expected plasma protein binding of 20%.^29^

The *fC*_ss_/MIC ratio of ceftolozane was selected as the only PD determinant of efficacy of ceftolozane/tazobactam therapy for the clinical and microbiological outcomes of severe *P. aeruginosa* infections. This choice aligned with the fact that the excellent activity of ceftolozane/tazobactam against *P. aeruginosa* is due exclusively to ceftolozane and is unaffected by tazobactam addition, irrespective of strains being WT or DTR.^7^ Optimal PK/PD target attainment was defined as an aggressive *fC*_ss_/MIC ratio of ceftolozane >4. Values between 1 and 4 and <1 were defined as quasi-optimal and suboptimal, respectively. In patients having multiple TDM-guided ECPAs, an average *fC*_ss_/MIC ratio of ceftolozane was calculated as the mean of all the *fC*_ss_/MIC ratios observed throughout the treatment course.

Microbiological eradication or failure were defined respectively as the eradication from or the persistence at the infection site of the index *P. aeruginosa* strain as documented by the follow-up cultures after more than 7 days from starting ceftolozane/tazobactam treatment.^30^ Resistance to ceftolozane/tazobactam of the index *P. aeruginosa* isolate was defined as an MIC increase beyond the EUCAST clinical breakpoint of susceptibility documented at any microbiological culture performed within 30 days.^30^ Clinical cure was defined as the complete resolution of signs and symptoms of infection coupled with documented microbiological eradication at end of treatment and absence of recurrence/relapse at 30 day follow-up and/or attributable mortality due to *P. aeruginosa* infection.^31^

Continuous data were described as median and IQR, whereas categorical variables were expressed as number and percentage. Univariate analysis comparing patients treated with CI ceftolozane/tazobactam in pre- versus post-intervention phases and those having microbiological eradication versus microbiological failure was performed by means of the Fisher exact test or the χ^2^ test (for categorial variables) or Mann–Whitney *U* test (for continuous variables). Multivariate logistic regression analysis was used to test variables potentially associated with microbiological failure in the two groups. The model was adjusted for age and gender in order to minimize the risk of potential confounders. Independent covariates having a *P* value  <0.20 at the univariate analysis were included in the multivariate logistic regression model. Statistical significance was identified by a *P* value  ≤0.05, namely having a 5% or less chance of incorrectly rejecting the null hypothesis when it is true. Statistical analysis was performed by means of MedCalc for Windows (MedCalc statistical software, version 19.6.1; MedCalc Software Ltd, Ostend, Belgium).

## Results

Overall, a total of 85 patients (48 in the pre-intervention phase and 37 in the post-intervention phase) were included in this study, whose demographics and clinical features are summarized in Table 1.

**Table 1.** Comparison of demographics and clinical characteristics of patients receiving CI ceftolozane/tazobactam monotherapy for documented *P. aeruginosa* infections

Variables	Overall (*n* = 85)	Pre-intervention phase: standard approach (*n* = 48)	Post-intervention phase: TDM-guided approach (*n* = 37)	*P* value
Demographics				
Age, median (IQR), y	67.0 (60.0–77.0)	68.0 (60.0–77.3)	63.0 (59.0–75.0)	0.50
Gender (male/female), *n* (%)	56/29 (65.9/34.1)	34/14 (70.8/29.2)	22/15 (59.5/40.5)	0.27
BMI, median (IQR), kg/m^2^	25.0 (21.9–28.3)	24.4 (21.4–28.1)	25.8 (23.7–29.3)	0.58
Obesity, *n* (%)	17 (20.0)	9 (18.8)	8 (21.6)	0.74
Underlying disease, *n* (%)				
Solid organ transplant recipient	17 (20.0)	8 (16.7)	9 (24.4)	0.38
Cardiac valve replacement	12 (14.1)	7 (14.6)	5 (13.5)	0.89
Vascular prosthesis placement	13 (15.3)	8 (16.7)	5 (13.5)	0.69
Haematological malignancies	11 (12.9)	6 (12.5)	5 (13.5)	0.89
Acute pulmonary oedema	7 (8.2)	4 (8.3)	3 (8.1)	0.99
Bowel perforation	5 (5.9)	3 (6.3)	2 (5.4)	0.99
Acute pancreatitis	5 (5.9)	3 (6.3)	2 (5.4)	0.99
Acute myocardial infarction	2 (2.4)	1 (2.1)	1 (2.7)	0.99
Others	13 (15.3)	8 (16.7)	5 (13.5)	0.69
Immunosuppression	38 (44.7)	21 (43.8)	17 (45.9)	0.84
Setting, *n* (%)				
ICU	63 (74.1)	32 (66.7)	31 (83.8)	0.07
Medical ward	14 (16.5)	11 (27.1)	3 (8.1)	0.08
Haematology	6 (7.1)	3 (6.3)	3 (8.1)	0.99
Surgical ward	2 (2.3)	2 (4.2)	0 (0.0)	0.50
Pathophysiological conditions				
SOFA score, median (IQR)	6 (3–9)	5 (3–9)	7 (3–9)	0.29
Vasopressors, *n* (%)	29 (34.1)	11 (22.9)	18 (48.6)	**0**.**01**
Mechanical ventilation, *n* (%)	49 (57.6)	24 (50.0)	25 (67.6)	0.10
Pao_2_/Fio_2_ ratio, median (IQR)	225 (150–316)	211.0 (156.0–312.5)	231.0 (128.8–304.0)	0.81
Pao_2_/Fio_2_ ratio < 200, *n* (%)	37 (43.5)	23 (47.9)	14 (37.8)	0.35
Baseline CL_CR_, median (IQR), mL/min/1.73 m^2^	69.0 (35.0–98.8)	79.0 (37.3–107.0)	62.5 (33.8–95.0)	0.27
Continuous renal replacement therapy, *n* (%)	15 (17.6)	9 (18.8)	6 (16.2)	0.76
Augmented renal clearance, *n* (%)	5 (5.9)	4 (8.3)	1 (2.7)	0.38
Site of infection, *n* (%)				
HAP/VAP	47 (55.3)	27 (56.3)	20 (54.1)	0.84
BSI	27 (31.8)	14 (29.2)	13 (35.1)	0.34
HAP/VAP + BSI	11 (12.9)	7 (14.5)	4 (10.8)	0.75
Late-onset VAP	33 (38.8)	22 (43.8)	11 (29.7)	0.13
*P. aeruginosa* phenotype, *n* (%)				
Multi-susceptible	50 (58.8)	27 (56.3)	23 (62.2)	0.58
DTR	35 (41.2)	21 (43.7)	14 (37.8)	0.58
Ceftolozane/tazobactam MIC, *n* (%)				
0.5 mg/L	25 (29.4)	10 (20.8)	15 (40.5)	**0**.**05**
1 mg/L	52 (61.2)	33 (68.8)	19 (51.4)	0.10
2 mg/L	4 (4.7)	2 (4.2)	2 (5.4)	0.99
4 mg/L	4 (4.7)	3 (6.2)	1 (2.7)	0.63
Ceftolozane treatment regimens				
Ceftolozane/tazobactam CI MD, median (IQR), g/d	6.0 g/3.0g (2.0 g/1.0g to 6.0 g/3.0 g)	6.0 g/3.0g (3.0 g/1.5g to 6.0 g/3.0 g)	3.0 g/1.5g (2.0 g/1.0g to 6.0 g/3.0 g)	0.06
Treatment duration, median (IQR), d	10.0 (7.0–13.0)	10.0 (7.0–13.5)	10.0 (8.0–13.0)	0.55
Outcome, *n* (%)				
Microbiological eradication^a^	43/65 (66.2)	18/32 (56.3)	25/33 (75.8)	0.10
30 day resistance development	13 (15.3)	9 (18.8)	4 (10.8)	0.37
Clinical cure	47 (55.3)	24 (50.0)	23 (62.2)	0.26
30 day mortality rate	20 (23.5)	10 (20.8)	10 (27.0)	0.79

Variables with statistical significane are reported in bold. BSI, bloodstream infection; CI, continuous infusion; DTR, difficult-to-treat resistant; HAP, hospital-acquired pneumonia; MD, maintenance dose; Pao_2_/Fio_2_, ratio of partial pressure of arterial oxygen to fraction of oxygen in the inhaled air; TDM, therapeutic drug monitoring; VAP, ventilator-associated pneumonia.

^a^32/48 and 33/37 patients had follow-up microbiological cultures in the pre- and post- intervention group, respectively.

Looking at the overall population, the median (IQR) age was 67 (60–77) years, with a male preponderance (65.9%). Solid organ transplantation was the most prevalent underlying disease (17 cases; 20.0%), followed by vascular prosthetic placement (15 cases; 15.3%), cardiac valve replacement (12 cases; 14.1%) and haematological malignancies (11 cases each; 12.9%). Sixty-three of 85 patients (74.1%) were ICU admitted, 49 needed mechanical ventilation (57.6%), 15 needed CRRT (17.6%) and 5 had ARC (5.9%). The median SOFA score was 6 (3–9).

HAP/VAP were the two most prevalent types of infection (47 cases; 55.3%), followed by BSI (27 cases; 31.8%) and HAP/VAP plus BSI (11 cases; 12.9%). Late-onset VAP occurred in 33 patients (38.8%). Among the 85 *P. aeruginosa* isolates, 50 were multi-susceptible (58.8%) and 35 were DTR (41.2%). All of the clinical isolates were susceptible to ceftolozane/tazobactam, with 77/85 (90.6%) having an MIC value  ≤1 mg/L.

By comparing the two groups, all the demographics and clinical characteristics of the patients were similar, apart from the need for vasopressors (48.6% versus 22.9%; *P* = 0.01) and the number of clinical isolates with an MIC value of 0.5 mg/L (40.5% versus 20.8%; *P* = 0.05), being significantly higher in the post-intervention group compared with the pre-intervention group. The median daily CI MD of ceftolozane/tazobactam trended to be higher in the pre-intervention group compared with the post-intervention group (6.0 g/3.0 g versus 3.0 g/1.5 g; *P* = 0.06).

In the post-intervention group, a total of 69 TDM-guided ECPAs of ceftolozane/tazobactam were provided, with a median (IQR) of 1 (1–2) per patient. At initial TDM-guided ECPAs (37/69), types of recommendations for dosage adjustments were decreases in 32/37 (86.5%) and increases in 2/37 (5.4%) of cases, respectively. Even at subsequent TDM-guided ECPAs (32/69), recommendations were mainly for decreases (20/32; 62.5%), with only a minority of increases (2/32; 6.2%). Optimal PK/PD target was always attained in all patients, with a median (IQR) *fC*_ss_/MIC ratio of 43.0 (24.6–79.3), and a median (IQR) ceftolozane *fC*_ss_ of 35.3 mg/L (21.0–76.2 mg/L).

Comparing outcomes, rates did not significantly differ between the pre-intervention versus the post-intervention group in terms of microbiological eradication (56.3% versus 75.8%; *P* = 0.10), 30 day resistance development to ceftolozane/tazobactam (18.8% versus 10.8%; *P* = 0.37), clinical cure (50.0% versus 62.2%; *P* = 0.26) and 30 day mortality rate (20.8% versus 27.0%; *P* = 0.79). The findings were confirmed even after sub-analysis according to the MIC distribution of the clinical isolates (0.5 or ≥1 mg/L; Table S1, available as Supplementary data at *JAC* Online).

Regression analysis assessing potential predictors associated with microbiological eradication versus failure of documented *P. aeruginosa* infections treated with CI ceftolozane/tazobactam monotherapy among patients having follow-up culture available in the pre-intervention (32/48) and post-intervention (33/37) phases are reported in Tables 2 and 3, respectively. At multivariate analysis, the only independent predictors of microbiological failure were DTR *P. aeruginosa* isolates in the pre-intervention group (OR 6.99; 95% CI 1.34–36.55; *P* = 0.02), and Pao_2_/Fio_2_ ratio  <200 in the post-intervention group (OR 18.00; 95% CI 1.86–174.22; *P* = 0.01).

**Table 2.** Univariate and multivariate analysis comparing patients having microbiological eradication versus microbiological failure treated with CI ceftolozane/tazobactam monotherapy for documented *P. aeruginosa* infections in the pre-intervention phase

Variables	Microbiological eradication (*n* = 18)	Microbiological failure (*n* = 14)	Univariate *P* value	Multivariate analysis^a^
Demographics				
Age, median (IQR)	71.0 (55.5–77.8)	73.0 (61.8–78.8)	0.44	
Gender (male/female), *n* (%)	13/5 (72.2/27.8)	9/5 (64.3/35.7)	0.71	
BMI, median (IQR)	23.0 (19.2–27.3)	24.0 (21.7–25.0)	0.72	
Obesity, *n* (%)	4 (22.2)	1 (7.1)	0.35	
Immunosuppression, *n* (%)	6 (33.3)	5 (35.7)	0.99	
Setting, *n* (%)				
ICU	11 (61.1)	9 (64.3)	0.99	
Medical ward	4 (22.2)	4 (28.6)	0.70	
Surgical ward	2 (11.1)	0 (0.0)	0.49	
Haematology	1 (5.6)	1 (7.1)	0.99	
Pathophysiological condition				
SOFA score, median (IQR)	5 (2–8.5)	5.5 (2.25–7)	0.80	
Vasopressors, *n* (%)	5 (27.8)	2 (14.3)	0.43	
Mechanical ventilation, *n* (%)	9 (50.0)	6 (42.9)	0.73	
Pao_2_/Fio_2_ ratio, median (IQR)	241.0 (150.0–369.5)	251.0 (186.3–332.0)	0.95	
Pao_2_/Fio_2_ ratio <200, *n* (%)	8 (44.4)	6 (42.9)	0.99	
Continuous renal replacement therapy, *n* (%)	3 (16.7)	2 (14.3)	0.99	
Augmented renal clearance, *n* (%)	3 (16.7)	0 (0.0)	0.24	
Site of infection, *n* (%)				
HAP/VAP	8 (44.4)	4 (28.6)	0.47	
BSI	8 (44.4)	6 (42.8)	0.99	
HAP/VAP + BSI	2 (11.2)	4 (28.6)	0.36	
Overall pneumonia	10 (55.6)	8 (57.2)	0.99	
Late-onset VAP	6 (33.3)	7 (50.0)	0.47	
*P. aeruginosa* susceptibility, *n* (%)				
Multi-susceptible	13 (72.2)	4 (28.6)	**0**.**03**	**0.027** **(OR 0.14; 95%CI 0.03–0.80)**
DTR	5 (27.8)	10 (71.4)	**0**.**03**	**0.021** **(OR 6.99; 95%CI 1.34–36.55)**
Ceftolozane/tazobactam MIC, *n* (%)				
=0.5 mg/L	4 (22.2)	4 (28.6)	0.70	
≥1 mg/L	14 (77.8)	10 (71.4)		

Variables with statistical significane are reported in bold. BSI, bloodstream infection; CI, continuous infusion; DTR, difficult-to-treat resistant; HAP, hospital-acquired pneumonia; Pao_2_/Fio_2_, ratio of partial pressure of arterial oxygen to fraction of oxygen in the inhaled air; VAP, ventilator-associated pneumonia.

^a^Multivariate analysis adjusted for age, gender and variables with *P* < 0.20 at univariate analysis.

**Table 3.** Univariate and multivariate analysis comparing patients having microbiological eradication versus microbiological failure treated with CI ceftolozane/tazobactam monotherapy for documented *P. aeruginosa* infections in the post-intervention phase

Variables	Microbiological eradication (*n* = 25)	Microbiological failure (*n* = 8)	Univariate *P* value	Multivariate analysis^a^
Demographics				
Age, median (IQR)	63.0 (59.0–72.0)	75.5 (62.0–79.3)	0.16	
Gender (male/female), *n* (%)	15/10 (60.0/50.0)	4/4 (50.0/50.0)	0.70	
BMI, median (IQR)	25.5 (22.4–26.6)	28.5 (25.4–29.8)	0.74	
Obesity, *n* (%)	5 (20.0)	2 (25.0)	0.99	
Immunosuppression, *n* (%)	14 (56.0)	3 (37.5)	0.44	
Setting, *n* (%)				
ICU	20 (80.0)	8 (100.0)	0.30	
Medical ward	3 (12.0)	0 (0.0)	0.56	
Haematology	2 (8.0)	0 (0.0)	0.99	
Pathophysiological condition				
SOFA score, median (IQR)	6 (3–8)	9 (8.25–11.25)	**0**.**048**	
Vasopressors, *n* (%)	9 (36.0)	6 (75.0)	0.10	
Mechanical ventilation, *n* (%)	14 (56.0)	8 (100.0)	**0**.**03**	
Pao_2_/Fio_2_ ratio, median (IQR)	257.0 (179.0–319.0)	125.0 (103.75–196.75)	**0**.**03**	
Pao_2_/Fio_2_ ratio <200, *n* (%)	7 (28.0)	7 (87.5)	**0**.**005**	**0.01** **(OR 18.00; 95% CI 1.86–174.22)**
Continuous renal replacement therapy, *n* (%)	3 (12.0)	3 (37.5)	0.14	
Augmented renal clearance, *n* (%)	1 (4.0)	0 (0.0)	0.99	
Site of infection, *n* (%)				
HAP/VAP	10 (40.0)	6 (75.0)	0.11	
BSI	12 (48.0)	1 (12.5)	0.11	
HAP/VAP + BSI	3 (12.0)	1 (12.5)	0.99	
Overall pneumonia	13 (52.0)	7 (87.5)	0.11	
Late-onset VAP	6 (24.0)	5 (62.5)	0.08	
*P. aeruginosa* susceptibility, *n* (%)				
Multi-susceptible	17 (68.0)	6 (75.0)	0.99	
DTR	8 (32.0)	2 (25.0)	0.99	
Ceftolozane/tazobactam MIC, *n* (%)				
=0.5 mg/L	10 (40.0)	5 (62.5)	0.42	
≥1 mg/L	15 (60.0)	3 (37.5)		
Ceftolozane treatment and PK/PD target attainment, *n* (%)				
Quasi-optimal/suboptimal PK/PD target attainment	0 (0.0)	0 (0.0)	0.99	
Ceftolozane *fC*_ss_/MIC, median (IQR)	45.3 (33.6–73.2)	25.4 (19.8–99.5)	0.58	

Variables with statistical significane are reported in bold.

BSI, bloodstream infection; CI, continuous infusion; DTR, difficult-to-treat resistant; *f*C_ss_, free steady-state concentration; HAP, hospital-acquired pneumonia; Pao_2_/Fio_2_, ratio of partial pressure of arterial oxygen to fraction of oxygen in the inhaled air; PK/PD, pharmacokinetic/pharmacodynamic; VAP, ventilator-associated pneumonia.

^a^Multivariate analysis adjusted for age, gender and variables with *P* < 0.20 at univariate analysis.

## Discussion

To the best of our knowledge, this is the first study that assessed, by means of a pre-post quasi-experimental study design, the potential impact of a TDM-guided ECPA programme focused on attaining an aggressive PK/PD target with CI ceftolozane/tazobactam monotherapy on the clinical and microbiological outcome of severe *P. aeruginosa* BSIs and/or pneumonia.

Overall, patients included in the two groups had very similar demographics and clinical characteristics. Interestingly, the rates of favourable clinical outcome and of 30 day resistance occurrence did not differ in the post-intervention phase from those observed in the pre-intervention phase, and the microbiological eradication rates tended to be higher in the post-intervention phase. In this regard, it is worth noting that the TDM-guided approach allowed us to achieve these goals in the post-intervention phase with ceftolozane/tazobactam dosages that tended to be significantly lower compared with those used in the pre-intervention phase. Since these dosages always allowed attainment of an aggressive PK/PD target against *P. aeruginosa* isolates, this means that the TDM-guided approach was helpful in lowering the ceftolozane/tazobactam dosages needed by CI for maximizing treatment efficacy when dealing with *P. aeruginosa* strains with an MIC value up to the clinical breakpoint of 4 mg/L.

These findings agree with previous preclinical and clinical studies showing the added value that CI administration of ceftolozane/tazobactam may have in decreasing the dosages needed for attaining aggressive PK/PD targets against infections caused by *P. aeruginosa* strains susceptible to ceftolozane/tazobactam.^14–16^ Specifically, a hollow fibre infection model against XDR *P. aeruginosa* isolates assessed the efficacy of decreased ceftolozane/tazobactam doses by CI (6 g/day) compared with standard doses either by extended infusion (3 g q8h over 3 h) or by intermittent infusion (3 g q8h over 1 h). Interestingly, 6 g/day by CI granted the largest reduction in bacterial density and was the only regimen granting bactericidal effect against all isolates, irrespective of whether they were susceptible (MIC of 2 mg/L) or resistant (MIC of 8–16 mg/L) to ceftolozane/tazobactam.^15^

In our patients having normal renal function the median *fC*_ss_ of ceftolozane after administering a median CI MD of 4.5 g/daily of ceftolozane/tazobactam was as high as 35.3 mg/L. This suggests a good likelihood of attaining an aggressive PK/PD target even against pathogens theoretically considered *in vitro* resistant to ceftolozane/tazobactam according to the EUCAST clinical breakpoint. Population PK studies would be needed for properly defining the best CI dosing regimens for attaining a properly aggressive PK/PD target. Meanwhile, it may be hypothesized that administering by CI the standard licensed dose for pneumonia, namely 9 g/day, could be helpful in attaining aggressive PK/PD targets even against *P. aeruginosa* strains with MIC values up to at least 8–16 mg/L. This could offer a potential opportunity for tipping the scales and optimizing PK/PD target attainment even against *P. aeruginosa* isolates theoretically resistant to ceftolozane/tazobactam.

On the other hand, for very susceptible clinical isolates the TDM-guided approach, by allowing reduction of the CI dosage needed for proper aggressive PK/PD target attainment, may also represent a valuable tool for preventing unnecessary overexposure associated with potentially dangerous excessive selective pressure. Curiously enough, the number of very susceptible *P. aeruginosa* isolates with an MIC of 0.5 mg/L approximately doubled in the post-intervention phase compared with the pre-intervention phase (40.5% versus 20.8%; *P* = 0.05). Additionally, it could represent a cost-effective strategy either for minimizing the potential toxicity risk deriving from unnecessary overexposure or for cost saving. Population PK studies would be warranted for properly defining the best CI dosing regimens ensuring the highest probability of attaining aggressive PK/PD targets. Overall, our approach aligns with recent guidance of the French Intensive Care Society for optimal use of novel β-lactams in critically ill patients.^32^

It is well known that *P. aeruginosa* infections caused by DTR strains can have greater clinical severity. In a retrospective observational study of 393 documented *P. aeruginosa* VAP episodes occurring in 314 patients, it was shown that MDR or XDR *P. aeruginosa* strains were independently associated with lower likelihood of ICU discharge without recurrence on Day 14 (*P* = 0.048).^33^ In our study DTR *P. aeruginosa*–related infection emerged as an independent predictor of microbiological failure but only in the pre-intervention phase. This finding might support the contention that optimizing aggressive PK/PD target attainment in the post-intervention group by means of the TDM-guided ECPA programme might have increased the likelihood of microbiological eradication of DTR *P. aeruginosa*–related infections.

Previous studies showed that respiratory failure and a low Pao_2_/Fio_2_ ratio in patients having VAP due to *P. aeruginosa* were associated with higher failure rate.^33,34^ A Pao_2_/Fio_2_ ratio <200 emerged as an independent predictor of microbiological failure only in the post-intervention phase. This might suggest that the added value of the TDM-guided approach in the post-intervention phase could have been limited when dealing with very severe cases of *P. aeruginosa* pneumonia.

Limitations of our study should be recognized. The retrospective monocentric study design and the limited sample size should be acknowledged. Although the overall impact of the patient population heterogeneity on the findings could not be ruled out, no type of ward admission setting was specifically associated with the risk of microbiological failure, either in the pre- or in the post-intervention phase. The absence of patients having only quasi-optimal/suboptimal PK/PD target attainment prevented us from testing this variable at the regression analysis as a potential predictor of microbiological failure in the post-intervention phase. The potential impact of selection bias on assessing microbiological outcome should be acknowledged since follow-up bronchoalveolar lavage cultures were unavailable in approximately one-quarter of cases. Any relationship between ceftolozane exposure and potential associated toxicity was not assessed. Susceptibility to ceftolozane was tested by the gold standard broth microdilution method as recommended by EUCAST, but potential intra-assay variability of the MIC value was not assessed. The prevalence of ARC in our population could be understated since CL_CR_ was not measured but only estimated. Finally, only total ceftolozane concentrations were measured, and the free fractions were calculated by considering fixed plasma protein binding retrieved in the literature without any individual correction based on the patient-specific plasma protein level. Conversely, the homogeneity of the demographics and clinical characteristics of the patients included in the two groups may represent a point of strength of our study.

In conclusion, the findings showed that our TDM-guided ECPA programme of CI ceftolozane/tazobactam was cost-effective in always attaining an aggressive PK/PD target of ceftolozane against susceptible *P. aeruginosa* strains with lower than standard daily doses but without compromising efficacy. In this scenario, the use of TDM-guided standard daily doses by CI could also represent an interesting option for raising the bar against theoretically resistant *P. aeruginosa* isolates. Larger prospective confirmatory studies are warranted.

## Supplementary Material

dkaf098_Supplementary_Data

## Transparency declarations

M. Gatti has received personal fees from Angelini and AdvanzPharma, outside the submitted work. P.V. has served as a consultant for bioMérieux, Gilead, Merck Sharp & Dohme, Nabriva, Nordic Pharma, Pfizer, Thermo-Fisher and Venatorx, and received payment for serving on the speakers’ bureau for Correvio, Gilead, Merck Sharp & Dohme, Nordic Pharma and Pfizer, outside the submitted work. F.P. participated in the speakers’ bureau for Angelini, Gilead, Menarini, InfectoPharm, Merck Sharp & Dohme, Pfizer, Shionogi and Thermo-Fisher, and on the advisory board for Advanz Pharma, Gilead, Merck Sharp & Dohme, Pfizer and Viatris, outside the submitted work. The other authors have no conflicts of interest to declare.

## Supplementary data

Table S1 is available as Supplementary data at *JAC* Online.
