# Supplementary material for: A pre-post quasi-experimental study of the impact of TDM-guided aggressive pharmacokinetic/pharmacodynamic target attainment of continuous infusion ceftolozane/tazobactam monotherapy in treating severe Pseudomonas aeruginosa infections: a strategy useful for raising the bar?
Source: J Antimicrob Chemother. 2025 Mar 24;80(6):1543–51. doi: 10.1093/jac/dkaf098 (PMC12129583; doi:10.1093/jac/dkaf098)
Supplement: dkaf098_Supplementary_Data [file dkaf098_supplementary_data.docx]

| **Supplementary Table 1 –** Univariate analysis comparing outcomes in the pre- and in the post-intervention cohort stratified by MIC distribution of clinical isolates. | | | | | | | | | | | | |
| --- | --- | --- | --- | --- | --- | --- | --- | --- | --- | --- | --- | --- |
|  | **Pre-intervention phase (n = 48)**  **Standard approach** | | | | | | **Post-intervention phase (n = 37)**  **TDM-guided approach** | | | | | |
|  | **No 30-day resistance development**  **(n=39/48)** | **30-day resistance development**  **(n=9/48)** | **p value** | **Clinical cure**  **(n=24/48)** | **Clinical failure**  **(n=24/48)** | **p value** | **No 30-day resistance development**  **(n=33/37)** | **30-day resistance development**  **(n=4/37)** | **p value** | **Clinical cure**  **(n=23/37)** | **Clinical failure**  **(n=14/37)** | **p value** |
| MIC = 0.5 mg/L | 8/48 (16.7) | 2/48 (4.2) | 0.99 | 5/48 (10.4) | 5/48 (10.4) | 0.99 | 13/37 (35.1) | 2/37 (5.4) | 0.99 | 7/37 (18.9) | 8/37 (21.6) | 0.11 |
| MIC ≥ 1 mg/L | 31/48 (64.5) | 7/48 (14.6) |  | 19/48 (39.6) | 19/48 (39.6) |  | 20/37 (54.1) | 2/37 (5.4) |  | 16/37 (43.2) | 6/37 (16.3) |  |
| TDM: therapeutic drug monitoring | | | | | | | | | | | | |
